# Lichen speciation is sparked by a substrate requirement shift and reproduction mode differentiation

**DOI:** 10.1038/s41598-022-14970-9

**Published:** 2022-06-30

**Authors:** Annina Kantelinen, Christian Printzen, Péter Poczai, Leena Myllys

**Affiliations:** 1 Botany Unit, Finnish Museum of Natural History, University of Helsinki, P.O. Box 7, 00014 Helsinki, Finland; 2 Senckenberg Forschungsinstitut und Naturmuseum Frankfurt, Senckenberganlage 25, 60325 Frankfurt am Main, Germany; 3 Museomics Research Group, Viikki Plant Science Centre (ViPS), University of Helsinki, PO Box 65, 00014 Helsinki, Finland

**Keywords:** Ecology, Evolution, Molecular biology

## Abstract

We show that obligate lignicoles in lichenized *Micarea* are predominately asexual whereas most facultative lignicoles reproduce sexually. Our phylogenetic analyses (ITS, mtSSU, *Mcm*7) together with ancestral state reconstruction show that the shift in reproduction mode has evolved independently several times within the group and that facultative and obligate lignicoles are sister species. The analyses support the assumption that the ancestor of these species was a facultative lignicole. We hypothezise that a shift in substrate requirement from bark to wood leads to differentiation in reproduction mode and becomes a driver of speciation. This is the first example of lichenized fungi where reproduction mode is connected to substrate requirement. This is also the first example where such an association is demonstrated to spark lichen speciation. Our main hypothesis is that obligate species on dead wood need to colonize new suitable substrata relatively fast and asexual reproduction is more effective a strategy for successful colonization.

## Introduction

Despite increased knowledge on lichen diversity, the factors influencing species richness and speciation are still largely unknown. Existing studies have mostly focused on extrinsic factors and found that diversification events are usually correlated with climatic changes such as climatic cooling events during the Tertiary^1^, aridification during the Oligocene–Miocene transition^2^, and Pleistocene glacial cycles^3^. Only few studies on lichens have considered extrinsic environmental factors and intrinsic lineage-specific traits jointly: Innovations in secondary chemistry (= extracellular products) together with a shift in substrate requirement were found to trigger adaptive radiation in the lichen family Teloschistaceae^4^. Increased nitrogen availability after acquisition of cyanobacterial symbionts led to an adaptive radiation in *Placopsis* (L.) Linds.^5^. Green-algal or cyanobacterial symbiont interactions through time and space may have influenced diversification in the genus *Sticta* (Schreb.) Ach.^6^. Interplay between intrinsic traits related to reproduction and extrinsic traits related to ecological opportunities are often correlated with shifts in species diversification in other organisms^7–10^ but this has never been examined in lichenized fungi.

Lichenized fungi have developed diverse reproduction strategies. Many have the ability to reproduce both sexually (ascospores) and asexually (conidia, thallus fragments i.e. soredia, isidia, goniocysts), while others are either sexual or asexual^11,12^. Diverse reproduction strategies are at least partly related to lichen symbiosis: asexual reproduction via thallus fragments ensures the continuation of symbiosis^13^, whereas successful sexual reproduction via ascospores requires that the germinating mycelium makes contact with a compatible free-living photobiont before the lichen thallus can develop^14–16^. One exception is the asexual propagules produced by the fungal partner, called conidia: they usually do not contain the photobiont, so they need to find a suitable one on the new substrate they land on^16^. However, both modes of asexual reproduction are assumed to typically consume less energy than sexual reproduction and do not rely on the availability of suitable mating partners. Therefore, asexual lichen lineages can be faster and more efficient at colonizing newly exposed substrates^17–19^. Recent studies have shown that asexual lineages are long-lived evolutionarily and can give rise to sexual lineages^11^.

**Figure 1:**
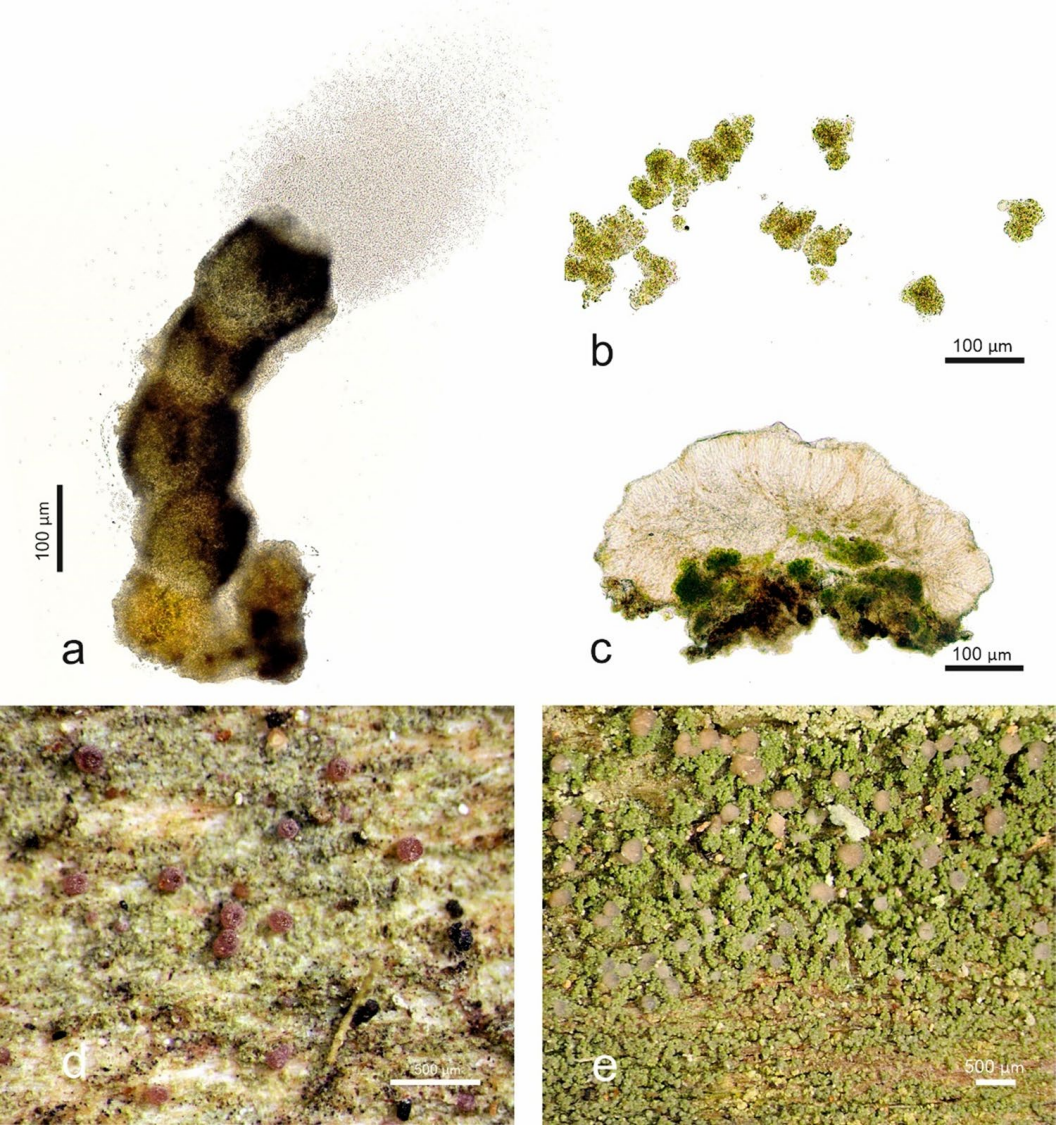
Sexual and asexual structures and reproduction strategies in the *Micarea prasina* group. (**a**) A pycnidium of *Micarea fennica* extruding asexual mesoconidia (Kantelinen 3220 holotype, H). Mesoconidia are small, likely easily carried by wind and insects and allow long-distance dispersal, (**b**) Thallus goniocysts of *M. hedlundii* including a mycobiont and a photobiont (Kantelinen 67119, H). Goniocysts are asexual vegetative structures that are relatively big and therefore probably more effective on short distance colonization, (**c**) Apothecial section of *M. microareolata* (Pykälä 47787, H). Sexual ascospores developed in apothecia are small, likely easily carried by wind and insects, and their development requires a mating partner and more energy than asexual diaspores, (**d**) Pycnidia and thallus of *M. tomentosa* (Kantelinen 29151, H), (**e**) Apothecia and thallus of *M. prasina* (Kantelinen 229106, H). Photos A. Kantelinen.

The microlichen genus *Micarea* is an excellent model for studying the effects of reproductive traits and environmental factors on speciation because it shows intricate variation in substrate requirements and reproduction modes. The genus is widespread worldwide and has lately received much scientific interest, resulting in over 20 new species descriptions^20–31^. Certain species are generalists able to grow on various substrata, while some are specialized and living in strict microhabitats^20,32–34^. A wide range of sexual and asexual propagules is found in *Micarea* (Fig. 1), including ascospores, three types of conidia (micro-, meso-, and macroconidia), and thallus fragments called goniocysts that likely act as asexual propagules including both symbiotic partners. The photobionts in the *M. prasina* group are small roundish green algae in the sister genera *Coccomyxa* and *Elliptochloris*^20,32,35^*.* Some of the *Micarea* species are predominately sexual, while some frequently lack sexual structures but bear numerous pycnidia where asexual conidia are produced. The actual roles of the three types of conidia present are not thoroughly understood, but mesoconidia are likely asexual propagules based on, for example, the observation that many of the species are frequently found with only mesopycnidia and no apothecia^20,27,32^.

Furthermore, *Micarea* represents one of the most important microlichen genera occurring on dead wood^36^. Of the 27 European species in the *M. prasina* group (a monophyletic “core group” including the type species), 17 are facultative lignicoles, five are obligate lignicoles, and only four have never been found on dead wood^20,24,27–30,36^. Of the facultative lignicoles, some species are often encountered on dead wood while others rarely occupy the substratum. Furthermore, some of the obligate lignicoles are rare and have very narrow ecological amplitudes, occurring only on wood of specific decay stages^20,27,34^.

In this study, our aim is to examine the reproduction modes and evolution of substrate preferences in the *M. prasina* group and how these features may affect speciation. We focus especially on asexual mesopycnidia and presence/absence of apothecia.

## Results

Altogether 516 herbarium specimens were studied. Each specimen was identified to species level by using relevant literature^20,21,23–25,27–30,32^, and the reproduction mode and substrate was recorded ("Appendix"). Our results confirm previous findings that obligate and facultative preference for dead wood are species-specific traits (see references above).

**Figure 2:**
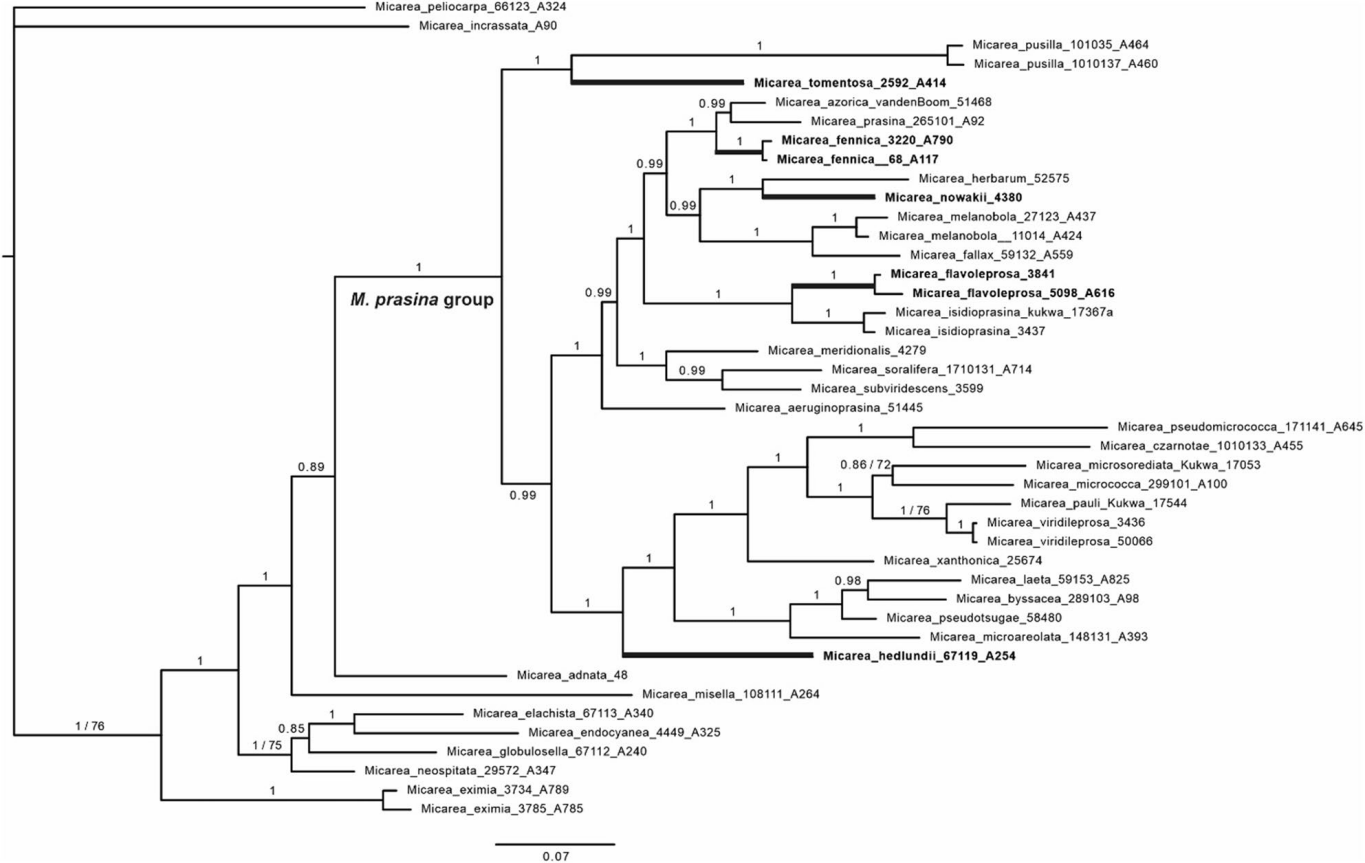
Bayesian tree based on concatenated sequences of ITS, mtSSU and *Mcm7*. Bayesian posterior probabilities are indicated above the nearest branches. Maximum likelihood values are marked if less than 80. Obligate lignicoles are marked in bold.

Representative specimens of each taxa were selected for phylogenetic reconstruction (Table 1). The analyses included three loci (ITS, *Mcm*7, mtSSU) and consisted of 110 sequences and of 1655 characters. The topologies of the Bayesian and maximum likelihood (ML) analyses did not show supported conflicts, and therefore only the tree obtained from the Bayesian analysis is shown (Fig. 2).

**Table 1:** List of *Micarea* specimens used in the phylogenetic analyses with locality, voucher information and GenBank accession numbers.

Taxon	Locality	Voucher information, sequence ID	ITS	mtSSU	*Mcm7*
*M. adnata*	Japan	*Andersen* 48 (BG)	AY756468	AY567751	–
*M. aeruginoprasina*	Portugal, Azores	*van den Boom* 51445 (LG), 3973	–	MK562024	MN105888
*M. azorica*	Portugal, Azores	*van den Boom* 51468 (LG), 3977	–	MK562026	MN105891
*M. byssacea*	Finland	*Launis* 289103 (H), A98	MG521562	MG707768	MG692527
*M. czarnotae*	Finland	*Launis* 1010133 (H), A455	MG521557	MG707760	MG692517
*M. elachista*	Finland	*Launis* 67113 (H), A340	MG521548	MG707745	–
*M. endocyanea*	USA, Maine	*Kantelinen* 4449 (H), A325	MT981601	MT982135	MT981445
*M. eximia*	Finland	*Kantelinen* 3785 (H), A785	MT981600	MT982134	MT981444
*M. eximia*	Finland	*Kantelinen* 3734 (H), A789	MT981599	MT982133	MT981443
*M. fallax*	Finland	*Launis* 59132 (H), A559	MK454942	MK454759	MK456617
*M. fennica*	Finland	*Launis* 3220 (H), A790	MK517712	MK517716	MK520931
*M. fennica*	Finland	*Launis* 68 (H), A117	MK517711	MK517715	MK520930
*M. flavoleprosa*	France	*Sérusiaux* s.n. (LG), 3841	–	MK454754	MK456613
*M. flavoleprosa*	Czech Republic	*Malíček* 5098 (H), A616	–	MK454756	MK456615
*M. globulosella*	Finland	*Launis* 67112 (H), A240	MG521546	MG707743	MG692507
*M. hedlundii*	Finland	*Launis* 67119 (H), A254	MG521551	MG707749	MG692512
*M. herbarum*	Netherlands	*P.* & *G. van den Boom* 52,575 (hb. van den Boom), LG DNA 4236	–	KX459349	MG692513
*M. incrassata*	Finland	*Kantelinen* 90 (H), A90	MT981598	MT982132	MT981442
*M. isidioprasina*	France	*Sérusiaux* s.n. (LG), 3437	MN095788	KX459362	MN105894
*M. isidioprasina*	Poland	*Kukwa* 17367a & *Łubek* (UGDA)	MN095789	MK562016	MN105897
*M. laeta*	Finland	*Launis* 59153 (H), A825	MG521565	MG707771	MG692530
*M. melanobola*	Finland	*Launis* 27123 (H), A437	MK454946	MK454770	MK456625
*M. melanobola*	Finland	*Launis* 11014 (H), A424	MK454950	MK454774	MK456630
*M. meridionalis*	Portugal	*van den Boom* s.n. (LG), 4279	–	KX459353	MN105901
*M. microareolata*	Sweden	*Launis* 148131 (H), A393	MG521558	MG707762	MG692518
*M. micrococca*	Finland	*Launis* 299101 (H), A100	MG521552	MG707753	MG692514
*M. microsorediata*	Poland	*Kukwa* 17053 (UGDA)	MN095791	MK562012	MN105906
*M. misella*	Finland	*Launis* 108111 (H), A264	MG521545	MG707742	MG692506
*M. neostipitata*	USA, North Carolina	*Lendemer* 29572 (H), A347	–	MT982136	–
*M. nowakii*	Romania	*Sérusiaux* s.n. (LG), 4380	–	KX459359	MN105908
*M. pauli*	Poland	*Kukwa* 17544 & *Łubek* (UGDA)	MN095795	MK562010	MN105913
*M. peliocarpa*	USA, Maine	*Launis* 66123 (H), A324	MG521544	MG707741	MG692505
*M. prasina*	Finland	*Launis* 265101 (H), A92	MG521549	MG707747	MG692510
*M. pseudomicrococca*	Scotland	*Launis* 171141 (H), A645	MG521556	MG707758	MG692516
*M. pseudotsugae*	Netherlands	*van den Boom 58480 UGDA*	–	MN547361	–
*M. pusilla*	Finland	*Launis* 1010137 (H), A460	MK454941	MK454752	MK456611
*M. pusilla*	Finland	*Launis* 101035 (H), A464	–	MK454753	MK456612
*M. soralifera*	Poland	*Kukwa* 13001 & *Łubek* (UGDA)	KT119887	KT119886	MN105917
*M. subviridescens*	Scotland	*Czarnota* 3599 (GPN)	–	EF453666	–
*M. tomentosa*	Finland	*Kantelinen* 2592 (H), A414	–	MT982138	MT981447
*M. viridileprosa*	Poland	*Czarnota* 3436 (GPN)	–	EF453671	–
*M. viridileprosa*	Netherlands	*P.* & *B. van den Boom*, 50066 (hb. van den Boom), LG DNA 3493	–	KX459366	MN105918
*M. xanthonica*	USA	*Tønsberg* 25674 (BG)	–	AY756454	–

**Figure 3:**
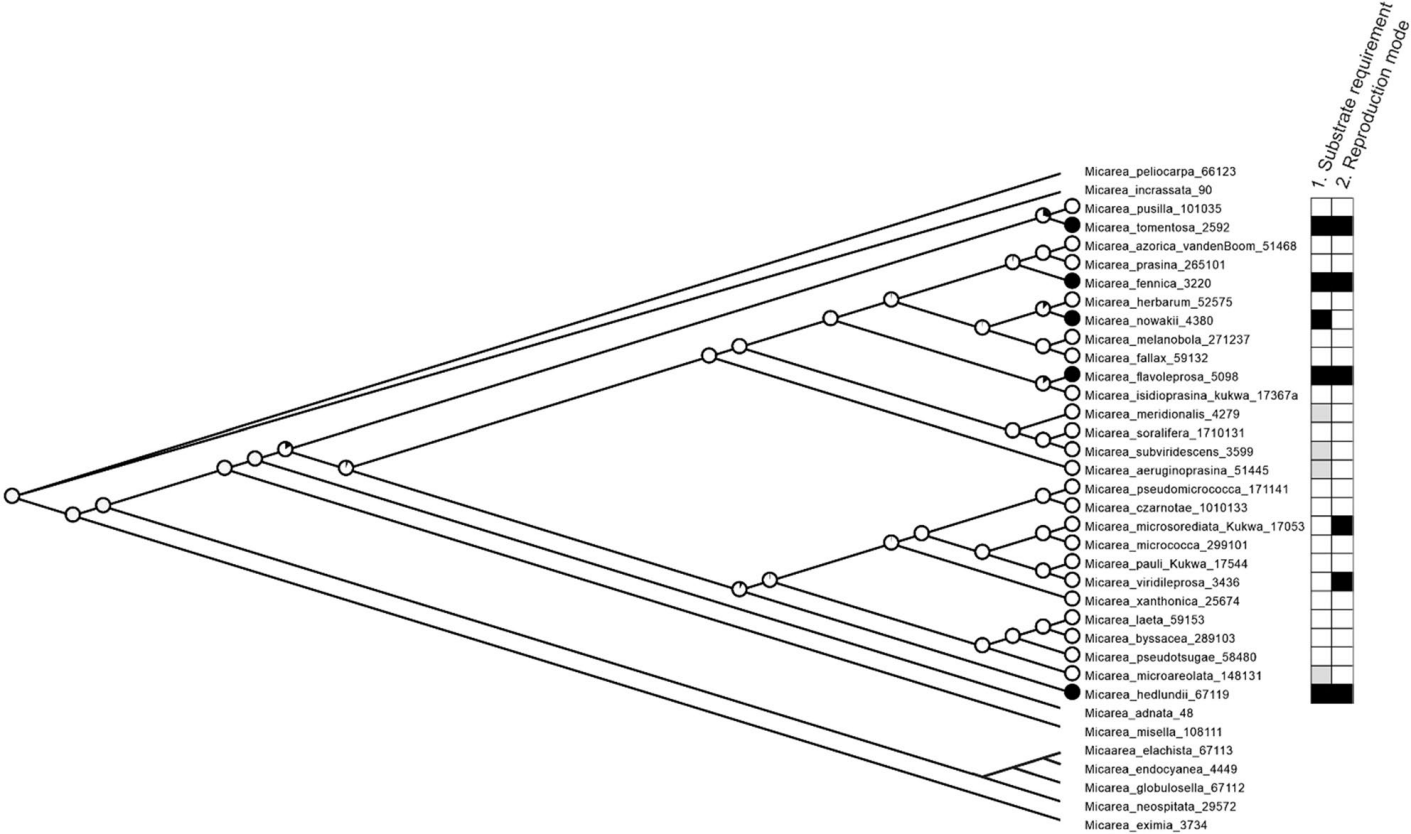
A maximum likelihood phylogram depicting ancestral character state reconstruction of the evolution of obligate lignicoles. Individuals of the same species were pruned and collapsed at the branches of the corresponding nodes. Pies represent probabilities of each ancestor being in two potential states for obligate lignicole (yes = black, no = white). In addition, substratum requirement and reproduction mode are mapped with black, grey and white boxes at the tips of the tree as follows: 1. Substratum requirement: black = obligate lignicole; white = facultative lignicole; grey = never found on dead wood. 2. Reproduction mode: black = predominately asexual; white = predominately sexual.

Our results show that obligate lignicoles occupying mid to late decay stages are predominately asexual (mesoconidia) while most facultative lignicoles reproduce sexually (ascospores) (Table 3). Our ancestral state reconstruction shows that the shift in reproduction mode has independently evolved several times within the group and that facultative and obligate lignicoles are sister species (Fig. 3). Furthermore, the ancestral state reconstructions support the ancestor of these species being a facultative lignicole. The statistic value of the correlation between reproductive mode and substrate preference is 0.0078, and the result was significant at *p* < 0.01 (Table 2). This connection was also indicated in Pagel’s test of correlated evolution since the difference (3.22) in the log-likelihood of both the four-parameter (− 27.25) and the eight-parameter model (− 24.03) showing presumably significant associations (*p* = 0.01 from 1000 simulations).

**Table 2:** Significance of the association between species´ reproduction modes and substrate preferences studied by Fisher Exact Test.

	Sexual	Asexual	Marginal Rows Totals
Obkigate lignicole	1	4	5
Facultative lignicole	16	2	18
Marginal column totals	17	6	23 (grand Total)

The test statistic value is 0.0078, and the result is significant at *p* < .01.

With strong support values, our phylogenetic reconstruction shows that lignicolous substratum preference has independently evolved several times within the group. Three out of the five obligate lignicoles are nested in the *M. prasina*-complex, i.e. *M. fennica*, *M. flavoleprosa*, and *M. nowakii*. *Micarea hedlundii* is resolved to be a separate lineage as a sister of the *M. byssacea* and *M. micrococca* complexes. *Micarea tomentosa* is resolved as a sister of the *M. byssacea*, *M. micrococca*, and *M. prasina* complexes. The 18 facultative lignicoles in our dataset are found in several lineages within the phylogeny.

## Discussion

Here, we report the *Micarea prasina* group as a first example of lichenized fungi where reproduction mode is connected to substrate preference. This is also the first example where such an association is demonstrated to be a driver of lichen speciation. Our study reveals that the shift in predominant reproduction mode has evolved independently several times within the group and that facultative and obligate lignicoles are sister species. Prior to our study, intrinsic and extrinsic factors involved in lichen speciation have rarely been studied jointly, and relationships between lichen substrate requirements and reproduction modes have not been deeply understood (but see^36,37^).

Many species in the *M. prasina* group are important colonizers of dead wood (eg.^20^). Spribille et al.^36^ concluded that most of the obligate lichen species growing on dead wood are sexually reproducing crustose lichens. Contrary to this view, our study shows that *Micarea* species occupying bark are predominantly sexual in their reproduction mode, whereas species restricted to dead wood reproduce asexually.

We believe there are several possible explanations for these obervations. Asexual reproduction on wood could be environmentally triggered. However, because facultative lignicoles form apothecia at normal frequencies on wood, the substrate itself cannot be the trigger. The fact that most obligately lignicolous lichens reproduce sexually^36^ further supports the notion that there are no intrinsic features of wood suppressing sexuality. Instead, we would have to assume another ecological trigger solely affecting four remotely related obligate lignicoles, but not their closest relatives or any other facultative lignicolous species in our data set, which we consider highly unlikely.

A more meaningful interpretation of our results involves the species’ life cycle. Species on decaying wood face a significant challenge as their substratum gradually changes and inevitably vanishes. When this happens, species need to colonize new suitable substrate. This may set a time limit, where asexual reproduction via mesoconidia, or with goniocysts acting as diaspores, is a faster and more effective strategy for successful colonization. Asexual lichen lineages are generally thought to be faster and more efficient at colonizing newly exposed substrates^17–19^. The complete decay of a log or stump can, however, take decades depending on the position of the tree^38^, and being restricted to an ephemeral substrate would only select for asexual reproduction if the generation time is long enough to effectively limit reproduction. Our results and literature show that many obligate lignicoles are restricted to certain decay stages^20,27,29,32^, which shortens the time frame they have for growth and reproduction^39,40^. *Micarea fennica*, *M. flavoleprosa*, *M. hedlundii*, and *M. tomentosa* mostly occur on late decay stages. *M. nowakii*, on the other hand, occupies hard wood in well-lit habitats. It is the only obligate lignicole in our data set that is predominately sexual (with additional mesoconidia nearly always abundantly present). This observation of *M. nowakii* can be explained by our hypothesis: selection would not work against sexual reproduction because the lengthy decay process does not impose a strict time limit for growth and reproduction.

The background of the speciation process suggested above is unknown, but a hypothetical population genetic scenario would be that generalist species experience lower fitness on wood than on bark. Selecting for traits that improve fitness on wood could have made the respective individuals or genotypes less competitive on bark. This kind of selection would ultimately lead to exclusive lignicoles alongside facultative ones.

In addition to our main hypothesis, the results could be explained by three alternative hypotheses, although we consider them as less likely. Our first alternative hypothesis is ecological: facultative lignicoles could experience lower fitness and produce less offspring on wood than on bark because of some inherent wood properties. Perhaps wood suppresses the formation of viable ascospores in *Micarea* (even if apothecia may still be produced) and being less dependent on ascospores for reproduction would lead to selection favoring asexual reproduction. Although logical, we do not have any evidence to support this theory. Our second alternative hypothesis is the rarity of dead wood: species in the *M. prasina* group could be heterothallic, and dead wood rarity in space and time could lead to fragmented and geographically isolated populations—Zoller et al*.*^41^ showed that such a situation could hinder the possibility for finding a mating partner, and sexual reproduction would therefore become unnecessary and rare. Plasticity in the reproduction of heterothallic species has previously been recorded in several lichenized fungal general^42–44^. However, despite this possibly explaining the prevalence of asexual reproduction in obligate lignicoles, it only holds true if populations on wood have already lost their capacity to live on bark. In other words, the development of asexual reproduction is only possible after speciation has already occurred. Substrate specialization could still lead to asexual reproduction but not be a driver of speciation. To date, we have no evidence to support this theory, e.g. *Micarea* specimens collected in areas with high dead wood densities are no more frequently sexual. In addition, the mating systems of *Micarea* have not been examined. Our third alternative hypothesis is closely linked to the previous one: studies (e.g. ^44^) have shown that lichens can reproduce asexually near the margins of their natural distribution while remaining sexual in central areas. This is usually because genetic diversity decreases towards margins making it difficult for heterothallic species to find mating partners. In theory, the obligate lignicoles in our data set could represent marginal populations of sexually reproducing species. So far, we have no evidence to support this theory, and we also consider it illogical that distribution would only affect obligate lignicoles, while facultative lignicoles would remain sexual.

Our data set includes two species that are predominately asexual but are not obligate lignicoles. *Micarea microsorediata* and *M. viridileprosa* occur on bark, wood, and on terrestrial substrates such as mosses. They rarely develop apothecia, and pycnidia have never been found from the latter. *Micarea microsorediata* mostly occurs in microhabitats where only few other lichen species co-exist, namely asexual *Lepraria*^25^. *Micarea viridileprosa*, on the other hand, develops widely spreading thallus that consists of small granules called goniocysts, and it appears to be an opportunistic species that is occasionally found on ephemeral substrates and growing over mosses and other lichens^20,45^. Based on our results, *Micarea* species that are not obligate lignicoles mostly reproduce sexually. However, the results on *M. microsorediata* and *M. viridileprosa* indicate that species occupying demanding low-light microhabitats or those that have opportunistic lifestyles benefit from asexual reproduction. Ecological strategies, niche requirements, and reproduction mode often correlate in other organisms (e.g.^46–49^).

## Conclusions

Museum collections have a pivotal role in shedding light on biological processes, as we have shown by analyzing a substantial amount of herbarium material. Natural history museums and herbaria remain a relatively untapped ’windows to the past’ in detecting, tracing and understanding non-model organisms.

Our results show that asexual reproduction is an evolutionary reaction on substrate specialization. Based on our preliminary results the relationship between asexual reproduction mode and wood-inhabiting lifestyle appears to exist beyond the *M. prasina* group, too. For example, *M. anterior*, *M. misella,* and the taxa in the *M. nigella* group are mostly found on dead wood and are predominately asexual^20,32,34^.

Future large-scale phylogenetic analyses could clarify how widespread the observed phenomenon is. With larger-scale data sets, aspects on diversification rates could also be addressed, that are difficult to tackle with our current data.

Finally, asexual reproduction in lichens is often regarded as a deficient version of sexual reproduction, but we encourage instead to view it as a gained ability and an advantageous lifestyle strategy.

## Methods

### Taxon sampling

Altogether 516 *Micarea* specimens were studied in the herbarium collections of FR, GPR, H, UPS and few specimens were also studied in LE, O and Hb Malíček ("Appendix"). We included all available specimens of relevant species into our data set. The studied specimens are collected from bark and dead wood by several collectors between 1940 and 2019. Older specimens from the 1800s and early 1900s were excluded from our study because asexually reproducing lichens were rarely collected at the time. Our data set includes *Micarea* specimens from the best known and widely collected areas in the world, i.e. Fennoscandia (Finland, Norway, Sweden) and Central Europe (Belarus, Czech Republic, the Netherlands, Germany, Poland, western Russia).

Since reliable information on species´ substrate requirements are crucial, we decided to leave out five species. Three of them, *Micarea pumila*, *M. stellaris* and *M. versicolor,* are newly described species from Kenya. During the Kenyan excursion specimens were looked for only from dead wood^31^. Another newly described species *Micarea nigra* is known based on only one collection from the Azores^25^. The fifth species, *M. levicula*, is known based on only three specimens from understudied areas in the tropics, and its substratum preferences and reproduction mode are poorly understood^31^. These five species are included in a previous contribution with a phylogeny^31^.

Reproduction structures (apothecia/mesopycnidia) and substratum (bark/dead wood/other) were recorded for each specimen ("Appendix"). The predominant reproduction mode and substrate requirement (facultative/obligate lignicole) were then calculated for each species using percentages (Table 3). Species with over 94% occurrence on dead wood were regarded as obligate lignicoles, *M. tomentosa* being the only such species with less than 100% occurrence on dead wood (one specimen from Poland is collected from decaying bark).

**Table 3:** Number of studied specimens, their reproduction mode and substrate requirement.

Species	No of studied specimens	No of speciemens with apothecia	No of speciemens with pycnidia	No of specimens on dead wood	No of specimens on bark	No of specimens on other substrata	% of specimens on dead wood	Substrate preference and predominant mode of reproduction
*M. aeruginoprasina*	4	4	rarely visible	0	4	0	0	Never on dead wood, sexual
*M. azorica*	4	4	rarely visible	0	4	0	0	Never on dead wood, sexual
*M. byssacea*	92	92	rarely visible	10	82	0	10, 9	Facultative lignicole, sexual
*M. czarnotae*	10	10	10	2	8	0	20	Facultative lignicole, sexual
*M. fallax*	52	52	rarely visible	46	7	0	86,8	Facultative lignicole, sexual
*M. fennica*	4	0	4	4	0	0	100	Obligate lignicole, asexual
*M. flavoleprosa*	9	1	5	9	0	0	100	Obligate lignicole, asexual
*M. hedlundii*	53	5	53	53	0	0	100	Obligate lignicole, asexual
*M. herbarum*	14	14	14	11	0	3	78,5	Facultative lignicole, sexual
*M. isidioprasina*	10	7 (few)	rarely visible	6	2	2	60	Facultative lignicole, sexual
*M. laeta*	19	19	rarely visible	2	17	0	10,53	Facultative lignicole, sexual
*M. melanobola*	24	24	rarely visible	9	15	0	37,5	Facultative lignicole, sexual
*M. meridionalis*	18	18	"often present"	0	18	0	0	Facultative lignicole, sexual
*M. microareolata*	9	9	rarely visible	0	9	0	0	Never on dead wood, sexual
*M. micrococca*	10	10	rarely visible	5	5	0	50	Facultative lignicole, sexual
*M. microsorediata*	29	4 (few)	none	7	22	0	24,1	Facultative lignicole, asexual
*M. nowakii*	17	17	14	17	0	0	100	Obligate lignicole, sexual
*M. pauli*	9	6	rarely visible	2	7	0	22,2	Facultative lignicole, sexual
*M. prasina*	43	43	rarely visible	38	5	0	88,37	Facultative lignicole, sexual
*M. pseudomicrococca*	23	23	rarely visible	14	9	0	60,86	Facultative lignicole, sexual
*M. pseudotsugae*	4	4	rarely visible	1	3	0	25	Facultative lignicole, sexual
*M. pusilla*	17	17	rarely visible	14	3	0	82,35	Facultative lignicole, sexual
*M. soralifera*	63	40	none	54	9	0	85,71	Facultative lignicole, sexual
*M. subviridescens*	9	8	rarely visible	0	0	9	0	Never on dead wood, sexual
*M. tomentosa*	17	5	17	16	1	0	94,12	Obligate lignicole, asexual
*M. viridileprosa*	29	Ap. rare	very rare	10	11	8	34,48	Facultative lignicole, asexual
*M. xanthonica*	41	20	not seen	not known	not known	not known	not known	Facultative lignicole, sexual

Five species are recorded based on literature: *M. herbarum* and *M. meridionalis*^23^, *M. subviridescens*(^53^ and NBN Atlas online records from herbarium E), *M. viridileprosa*^45^ and *M. xanthonica*^50^. Many *Micarea* species produce mesopycnidia that are immersed between goniocysts and may therefore be rarely visible.

Relevant literature on species ecology was also studied^20,21,23–25,27–32,34,45,50–52^, and our results on substrate preferences and reproduction modes are in line with the literature cited. Some of the specimens studied by previous authors are not included in our data set, however, because substrate and reproduction mode for the specimen is not reported.

### Morphology and chemistry

Each specimen in the data set was carefully studied and identified. Specimens were initially studied using dissecting microscopes (Leica S4E, ZEISS Stemi SV 11). Anatomical features were then examined on hand-cut apothecial sections and squash preparations mounted in water using compound microscopes (Leica CME, ZEISS Axioskop plus microscope). Ascospore dimensions and other anatomical measurements were made in water and in potassium hydroxide (K). Chemical spot tests were performed under a compound microscope using sodium hypochlorite (C) and 10% (K) to study the secondary chemistry and pigments^54^. Pigments were defined following Coppins^32^, Meyer & Printzen^55^, and Czarnota^20^. Some specimens were further studied using thin-layer chromatography (solvent C) following Culberson & Kristinsson^56^ and Orange et al.^54^. The crystalline granules of selected specimens were investigated using compound microscopes with polarization lenses.

### DNA extraction, polymerase chain reaction, and DNA sequencing

The sequences used in this study have been prepared during our previous studies (e.g. ^29,31^). Genomic DNA was extracted from 1–3 apothecia of specimens stored for a maximum of one year, using the DNeasy Blood & Tissue Kit (Qiagen, Maryland, USA) following the protocol described by Myllys et al. ^57^. Polymerase chain reactions (PCRs) were prepared using PuReTaq Ready-To- Go PCR Beads (GE Healthcare, Chicago, Illinois, USA). Each 25-μl reaction volume contained 19 μl distilled water (dH2O), 1 μl of each primer (10 μM), and 4 μl extracted DNA. The primers listed below were used for PCR amplification and sequencing. For the ITS region, PCR was run under the following conditions: initial denaturation for 5 min at 95 °C followed by five cycles of 30 s at 95 °C (denaturation), 30 s at 58 °C (annealing), and 1 min at 72 °C (extension); for the remaining 40 cycles, the annealing temperature was decreased to 56 °C; the PCR program ended with a final extension for 7 min at 72 °C. The primers used were ITS1-LM^58^ and ITS4^59^. For the mtSSU gene, PCR was run under the following conditions: initial denaturation for 10 min at 95 °C followed by six cycles of 1 min at 95 °C (denaturation), 1 min at 62 °C (annealing), and 1 min 45 s at 72 °C (extension); for the remaining 35 cycles, the annealing temperature was decreased to 56 °C; the PCR program ended with a final extension of 10 min at 72 °C. The primers used were mrSSU1 and mrSSU3R^60^. For the *Mcm*7 gene, PCR was run under two different conditions depending on the primers selected. For the first protocol, initial denaturation for 10 min at 94 °C was followed by 38 cycles of 45 s at 94 °C (denaturation), 50 s at 55 °C (annealing), and 1 min at 72 °C (extension), with the PCR program ending with a final extension for 5 min at 72 °C. The primers used were MCM7_AL1r and MCM7_AL2f.^27^. The second protocol used an initial denaturation for 10 min at 94 °C, followed by 38 cycles of 45 s at 94 °C (denaturation), 50 s at 56 °C (annealing), and 1 min at 72 °C (extension); the PCR program ended with a final extension for 5 min at 72 °C. The primers used were x.Mcm7.f^61^ and Mcm7.1348R^62^. PCR products were cleaned and sequenced by Macrogen Inc. (Amsterdam, The Netherlands; www.macrogen.com).

### Phylogenetic analyses

Phylogenies comprising 29 ITS, 44 mtSSU, and 37 *Mcm*7 sequences were first aligned separately with MUSCLE v.3.8.31^63^ using the European Molecular Biology Laboratory, European Bioinformatics Institute’s (EMBL-EBI) freely available web server (http://www.ebi.ac.uk/Tools/msa/muscle/). The single gene trees did not show any strongly supported conflicts according to the approach of Kauff & Lutzoni^64^ (with threshold bootstrap values ≥ 75%), and the three data sets were combined into a concatenated matrix in PhyDE (Phylogenetic Data Editor, http://www.phyde.de/index.html). Based on our previous studies^28,29^ and our preliminary phylogenetic reconstruction, *Micarea incrassata* Hedl*.* and *M. peliocarpa* (Anzi) Coppins & R. Sant. were used as outgroups. The hypervariable region at the end of the mtSSU and the ambiguously aligned region at the end of the ITS2 were removed from the analyses. The concatenated data set, including 44 terminals, was subjected to Bayesian inference using MrBayes (v. 3.2.7a)^65^ and to maximum likelihood (ML) analysis using RAxML 8.1.15^66^. For the Bayesian analysis, substitution models were selected by having the MCMC procedure sample across models^67^. The convergence of the four parallel runs was checked after 600 000 generations (sample freq 250, print freq 250) using Tracer (v. 1.5)^68^ and graphed using FigTree (v. 1.4.4). For the ML analysis, the combined data set was assigned to seven partitions: ITS1, 5.8S, ITS2, mtSSU, and each of the three codon positions of *Mcm*7. An independent GTR + G model was used for each subset, and branch lengths were assumed to be proportional across subsets. Node support was estimated with 1000 bootstrap replicates using the rapid bootstrap algorithm. The alignments are available from the Dryad Digital Repository (https://doi.org/10.5061/dryad.w9ghx3frx).

### Ancestral state reconstruction

A binary matrix was prepared with character states given for each taxon (obligate lignicole: yes/no). Reconstructions were made with Mesquite v3.40^69^ using parsimony and maximum likelihood methods.

In addition, substratum requirement and reproduction mode were studied by mapping states at the tips of the tree based on calculations in Table 3. Substratum requirement was mapped as: 1. obligate lignicole; 2. facultative lignicole; 3. neither. Predominant reproduction mode was mapped as: 1. asexual, i.e. mesopycnidia present and often numerous, apothecia rare or absent; 2. sexual, i.e. apothecia present and often abundant, mesopycnidia sometimes present (in some cases mesopycnidia may be present but invisible). We consider species predominately asexual when they do not develop apothecia (or do so rarely and only a few, eg. 1–3 per specimen), and produce mesopycnidia and -conidia. Goniocysts, on the other hand, are developed by nearly all species in the *M. prasina* group.

The alignments are available from the Dryad Digital Repository (https://doi.org/10.5061/dryad.w9ghx3frx).

### Character evolution analysis

A Fisher´s Exact Test was performed for our data to test if the association between species´ reproduction modes and substrate preferences are significant across the studied species. In addition, Pagel’s test^70^ of correlated character evolution was calculated for the *Micarea prasina* group from 10 iterations and 1000 replicates for simulations implemented in Mesquite v3.40. The effect of shifts between being obligate lignicole to/from a generalist, defined as all other species found on wood plus other substrates was also tested.

## Supplementary Information

Supplementary Information.
