# Supplementary material for: Lichen speciation is sparked by a substrate requirement shift and reproduction mode differentiation
Source: Sci Rep. 2022 Jun 30;12:11048. doi: 10.1038/s41598-022-14970-9 (PMC9247095; doi:10.1038/s41598-022-14970-9)
Supplement: Supplementary file 1 — Supplementary Information. [file 41598_2022_14970_MOESM1_ESM.pdf]

Appendix Studied 516 herbarium specimens in FR, GPR, H, LE, O, UPS and Hb Malíček. Reproduction structures (apothecia/mesopycnidia) and substratum (bark/dead wood/other), collector and herbarium number are marked for each specimen.

Species	Reproduction structures			Substratum			Collection country	Collector	Voucher number	Herbarium
	Apothecia	Mesopycnidia (if visible)	Goniocysts or soralia	Wood	Bark	Other				
Micarea byssacea	x		x		x		Poland	Kowalewska	UGDA-L-26393	UGDA
Micarea byssacea	x		x		x		Poland	Kowalewska	UGDA-L-26354	UGDA
Micarea byssacea	x		x		x		Poland	Kukwa	19988	UGDA
Micarea byssacea	x		x		x		Poland	Kukwa	19210	UGDA
Micarea byssacea	x		x		x		Poland	Kukwa	17362	UGDA
Micarea byssacea	x		x		x		Poland	Kukwa	17031	UGDA
Micarea byssacea	x		x		x		Poland	Kowalewska	UGDA-L-23026	UGDA
Micarea byssacea	x		x		x		Poland	Kukwa	UGDA-L-22707	UGDA
Micarea byssacea	x		x		x		Poland	Kukwa	17130	UGDA
Micarea byssacea	x		x	x			Poland	Kukwa	15612a	UGDA
Micarea byssacea	x		x		x		Poland	Kukwa	15605	UGDA
Micarea byssacea	x		x	x			Poland	Kukwa	15582	UGDA
Micarea byssacea	x		x	x			Poland	Kukwa	17233	UGDA
Micarea byssacea	x		x		x		Poland	Kukwa	14157	UGDA
Micarea byssacea	x		x		x		Poland	Kukwa	15760	UGDA
Micarea byssacea	x		x		x		Poland	Kukwa	15782	UGDA
Micarea byssacea	x		x		x		Poland	Kukwa	15953	UGDA
Micarea byssacea	x		x		x		Poland	Kukwa	12873	UGDA
Micarea byssacea	x		x		x		Poland	Kukwa	8315	UGDA
Micarea byssacea	x		x	x			Poland	Kukwa	12975	UGDA
Micarea byssacea	x		x		x		Poland	Kukwa	12626	UGDA
Micarea byssacea	x		x		x		Poland	Kukwa	12084	UGDA
Micarea byssacea	x		x		x		Poland	Kukwa	8318	UGDA
Micarea byssacea	x		x		x		Poland	Kukwa	UGDA-L-18880	UGDA

Micarea byssacea	x		x		x		Poland	Kukwa	8158	UGDA
Micarea byssacea	x		x		x		Poland	Kukwa	13519	UGDA
Micarea byssacea	x		x		x		Poland	Kukwa	UGDA-L-21303	UGDA
Micarea byssacea	x		x		x		Poland	Kukwa	13705	UGDA
Micarea byssacea	x		x		x		Poland	Kowalewska	UGDA-L-21822	UGDA
Micarea byssacea	x		x		x		Poland	Kowalewska	UGDA-L-21386	UGDA
Micarea byssacea	x		x		x		Poland	Kwiatkowska	UGDA-L-23984	UGDA
Micarea byssacea	x		x		x		Poland	Kukwa	17433	UGDA
Micarea byssacea	x		x		x		Poland	Kukwa	17435	UGDA
Micarea byssacea	x		x	x			Germany	Schneider	FR-0262918	FR
Micarea byssacea	x	x	x		x		Germany	Printzen & students	TS2-88-2	FR
Micarea byssacea	x		x		x		Germany	Printzen & students	TS2-88-13	FR
Micarea byssacea	x		x		x		Germany	Printzen & students	TS2-88-16-6	FR
Micarea byssacea	x		x		x		Germany	Printzen & students	TS2-99-3-1	FR
Micarea byssacea	x		x	x	x		Germany	Printzen & students	TS2-88-19-16	FR
Micarea byssacea	x		x	x			Germany	Printzen & students	TS2-88-16_20	FR
Micarea byssacea	x		x	x			Germany	Printzen & students	TS2-88-16_11	FR
Micarea byssacea	x		x		x		Germany	Printzen & students	TS2-99-7_12	FR
Micarea byssacea	x		x	x			Germany	Printzen & students	TS2-99-9_15	FR
Micarea byssacea	x		x		x		Germany	Printzen & students	TS2-99-10_14	FR
Micarea byssacea	x		x		x		Germany	Printzen & students	TS2-99-10_22	FR
Micarea byssacea	x	x	x		x		Sweden	Nordin, Sundin & Thor	(L-161761)365715	UPS
Micarea byssacea	x	(x)	x		x		Sweden	Nordin	L-917462	UPS
Micarea byssacea	x		x	x	x		Sweden	Nordin	L-891109	UPS
Micarea byssacea	x		x		x		Sweden	Westberg, Ekman, Hirschheydt	L-872309	UPS
Micarea byssacea	x		x		x		Sweden	Westberg, Ekman, Hirschheydt	L-872172	UPS

Micarea byssacea	x		x		x		Sweden	Nordin	(L-202817)506146	UPS
Micarea byssacea	x		x		x		Sweden	Ekman, Westberg, Svensson, Hirschheydt	L-872214	UPS
Micarea byssacea	x		x		x		Sweden	Nordin	L-797414	UPS
Micarea byssacea	x		x	x			Sweden	Westberg	L-950851	UPS
Micarea byssacea	x		x		x		Finland	Pykälä	53268	H
Micarea byssacea	x	x	x		x		Finland	Pykälä	53170	H
Micarea byssacea	x		x		x		Finland	Pykälä	53067	H
Micarea byssacea	x		x		x		Finland	Pykälä	53083	H
Micarea byssacea	x		x		x		Finland	Pykälä	53074	H
Micarea byssacea	x		x		x		Finland	Pykälä	54169	H
Micarea byssacea	x		x		x		Finland	Pykälä	53273	H
Micarea byssacea	x		x		x		Finland	Pykälä	53278	H
Micarea byssacea	x		x		x		Finland	Launis (Kantelinen)	289101	H
Micarea byssacea	x		x		x		Finland	Launis (Kantelinen)	289102	H
Micarea byssacea	x		x		x		Finland	Launis (Kantelinen)	289104	H
Micarea byssacea	x		x		x		Finland	Launis (Kantelinen)	208121	H
Micarea byssacea	x		x		x		Sweden	Czarnota	Czarnota s.n.	H
Micarea byssacea	x		x		x		Finland	Pykälä	47782	H
Micarea byssacea	x		x		x		Finland	Pykälä	47789	H
Micarea byssacea	x		x		x		Finland	Pykälä	47784	H
Micarea byssacea	x		x		x		Finland	Pykälä	47788	H
Micarea byssacea	x		x		x		Russia	Himelbrant & Stepanchikova	H9220198	H
Micarea byssacea	x		x		x		Finland	Thor	9445	UPS
Micarea byssacea	x		x		x		Finland	Kantelinen	4670	H

Micarea byssacea	x		x		x		Germany	Schneider	FR-0263061	FR
Micarea byssacea	x		x		x		Germany	Schneider	FR-0263020	FR
Micarea byssacea	x		x		x		Sweden	Mattsson	(L-95673)167568	UPS
Micarea byssacea	x		x		x		Germany	Schneider	FR-0263356	FR
Micarea byssacea	x		x		x		Germany	Schneider	FR-0263209	FR
Micarea byssacea	x		x		x		Sweden	Knutsson	L-720685	UPS
Micarea byssacea	x		x		x		Sweden	Knutsson	L-724517	UPS
Micarea byssacea	x		x		x		Sweden	Arup, Ekman & Knutsson	L-720671	UPS
Micarea byssacea	x		x		x		Sweden	Knutsson	L-724621	UPS
Micarea byssacea	x		x		x		Sweden	Knutsson	L-724617	UPS
Micarea byssacea	x		x		x		Sweden	Tibell	L-726862	UPS
Micarea byssacea	x		x		x		Sweden	Nordin, Sundin & Thor	(L-161767)365721	UPS
Micarea byssacea	x		x		x		Sweden	Nordin, Sundin & Thor	(L-161760)365714	UPS
Micarea byssacea	x		x		x		Sweden	Nordin, Sundin & Thor	(L-161763)365717	UPS
Micarea byssacea	x		x		x		Sweden	Thor	(L-174172)434195	UPS
Micarea byssacea	x		x		x		Sweden	Thor	L-745658	UPS
Micarea byssacea	x		x		x		Sweden	Svensson	(L-167556)399878	UPS
Micarea byssacea	x		x		x		Sweden	Thor	(L-174170)434193	UPS
Micarea byssacea	x		x	x			Germany	Schneider	FR-0263041	FR
Micarea czarnotae	x	x	x		x		Finland	Launis (Kantelinen)	109111	H
Micarea czarnotae	x	x	x	x			Finland	Launis (Kantelinen)	1010133	H
Micarea czarnotae	x	x	x	x			Netherland	van den Boom	50312	LG
Micarea czarnotae	x	x	x		x		Poland	Czarnota	3632	GPN
Micarea czarnotae	x	x	x		x		Poland	Czarnota	4179	GPN

Micarea czarnotae	x	x	x		x		Poland	Czarnota	3179	GPN
Micarea czarnotae	x	x	x		x		Poland	Czarnota	4059	GPN
Micarea czarnotae	x	x	x		x		Russia	Himelbrant & Stepanchikova	H9219944	H
Micarea czarnotae	x	x	x		x		Sweden	Nordin, Sundin & Thor	(L-161768)365722	UPS
Micarea czarnotae	x	x	x		x		Sweden	Nordin, Sundin & Thor	(L-161757)365711	UPS
Micarea fallax	x		x	x			Germany	Schneider	FR-0263156	FR
Micarea fallax	x		x	x			Germany	Schneider	FR-0263079	FR
Micarea fallax	x		x	x			Germany	Printzen & students	TS3-2,-15	FR
Micarea fallax	x		x	x			Germany	Printzen & students	TS3-15,-14	FR
Micarea fallax	x		x	x			Germany	Printzen & students	TS3-54,-7	FR
Micarea fallax	x		x	x			Germany	Printzen & students	TS3-17,-16	FR
Micarea fallax	x		x	x			Germany	Printzen & students	TS3-49,-9	FR
Micarea fallax	x		x	x			Germany	Printzen & students	TS3-35,-11	FR
Micarea fallax	x		x	x			Germany	Printzen & students	TS3-39,-13	FR
Micarea fallax	x		x	x			Germany	Printzen & students	TS3-41,-10	FR
Micarea fallax	x		x	x			Germany	Printzen & students	TS3-41,-11	FR
Micarea fallax	x		x	x			Germany	Printzen & students	TS3-37,-11	FR
Micarea fallax	x		x	x			Germany	Printzen & students	TS3-48,-13	FR
Micarea fallax	x		x	x			Germany	Printzen & students	TS3-48,-4	FR
Micarea fallax	x		x	x			Germany	Printzen & students	TS4-26,-3	FR
Micarea fallax	x		x	x			Germany	Printzen & students	TS4-21,-10	FR
Micarea fallax	x		x	x			Germany	Printzen & students	TS2-88-6	FR
Micarea fallax	x		x	x			Germany	Printzen & students	TS2-88-16-6	FR
Micarea fallax	x		x	x			Germany	Printzen & students	TS4-21,-11	FR
Micarea fallax	x		x	x			Germany	Printzen & students	TS4-60	FR
Micarea fallax	x		x	x			Germany	Printzen & students	TS4-40,-10	FR
Micarea fallax	x		x	x			Germany	Printzen & students	TS4-36,-6	FR

Micarea fallax	x		x	x			Germany	Printzen & students	TS4-53,-12	FR
Micarea fallax	x		x	x			Germany	Printzen & students	TS4-5,-13	FR
Micarea fallax	x		x	x			Germany	Printzen & students	TS4-23,-6	FR
Micarea fallax	x		x	x			Germany	Printzen & students	TS4-40,-10	FR
Micarea fallax	x		x	x			Germany	Printzen & students	TS4-1,-5	FR
Micarea fallax	x		x	x			Germany	Printzen & students	TS4-1,-12	FR
Micarea fallax	x		x	x			Germany	Printzen & students	TS4-16,-11	FR
Micarea fallax	x		x	x			Germany	Printzen & students	TS4-2,-10	FR
Micarea fallax	x		x	x			Germany	Printzen & students	TS4-20,-8	FR
Micarea fallax	x		x	x			Germany	Printzen & students	TS3-57,-4	FR
Micarea fallax	x		x	x			Germany	Printzen & students	TS4-29,-11	FR
Micarea fallax	x		x	x			Germany	Printzen & students	TS4-20,-9	FR
Micarea fallax	x		x	x			Germany	Printzen & students	TS4-20,-14	FR
Micarea fallax	x		x	x			Germany	Printzen & students	TS3-28,-8	FR
Micarea fallax	x		x	x			Germany	Printzen & students	TS3-46,-15	FR
Micarea fallax	x		x	x			Germany	Printzen & students	TS4-30,-13	FR
Micarea fallax	x		x		x		Finland	Launis (Kantelinen)	109115	H
Micarea fallax	x		x		x		Belarus	Tsurykau	001c4	H
Micarea fallax	x		x		x		Czech	Malíček	6127	Hb Malíček
Micarea fallax	x		x	x			Czech	Malíček	11821	Hb Malíček
Micarea fallax	x		x	x			Czech	Malíček	11992	Hb Malíček
Micarea fallax	x		x		x		Finland	Launis (Kantelinen)	27122	H
Micarea fallax	x		x	x			Finland	Launis (Kantelinen)	59132	H
Micarea fallax	x		x		x		Finland	Launis (Kantelinen)	1710132	H
Micarea fallax	x		x	x			Finland	Launis (Kantelinen)	1010138	H
Micarea fallax	x		x	x			Finland	Launis (Kantelinen)	1010139	H

Micarea fallax	x		x	x			Sweden	Svensson	2398	H
Micarea fallax	x		x	x			Russia	Himelbrant, Konoreva & Stepanchikova	GL-19	H
Micarea fallax	x		x		x		Sweden	Ågren	545	UPS
Micarea fallax	x		x		x		Poland	Kukwa	13639	UGDA
Micarea fennica		x	x	x			Finland	Launis (Kantelinen)	3220	H
Micarea fennica		x	x	x			Finland	Launis (Kantelinen)	68	H
Micarea fennica		x	x	x			Norway	Klepsland	?	O
Micarea fennica		x	x	x			Norway	Klepsland	?	O
Micarea flavoleprosa			x	x			Poland	Kukwa	14048	UGDA
Micarea flavoleprosa	x		x	x			Poland	Kukwa	14244	UGDA
Micarea flavoleprosa		x	x	x			Poland	Kukwa	15582a	UGDA
Micarea flavoleprosa	(x)		x	x			Czech	Malíček	5098	PRA
Micarea flavoleprosa			x	x			Austria	Berger	32900	Hb Berger
Micarea flavoleprosa			x	x			Austria	Berger	33573	Hb Berger
Micarea flavoleprosa			x	x			Czech	Malíček	4699	Hb Malíček
Micarea flavoleprosa			x	x			Czech	Malíček	11823	Hb Malíček
Micarea flavoleprosa			x	x			France	Sérusiaux	s.n.	LG
Micarea flavoleprosa		x	x	x			Poland	Kukwa	3168	UGDA
Micarea hedlundii		x	x	x			Poland	Czarnota	UGDA-L-9340	UGDA
Micarea hedlundii		x	x	x			Poland	Kukwa	17364	UGDA
Micarea hedlundii		x	x	x			Poland	Kukwa	17375	UGDA
Micarea hedlundii		x	x	x			Sweden	Weibull	L-739958	UPS
Micarea hedlundii		x	x	x			Sweden	Muhr	(L-08810) 23700	UPS
Micarea hedlundii		x	x	x			Sweden	Nordin	(L-64015)113111	UPS
Micarea hedlundii		x	x	x			Sweden	Muhr	L-591888	UPS

Micarea hedlundii		x	x	x			Sweden	Hermansson	(L-173108)429990	UPS
Micarea hedlundii	x	x	x	x			Sweden	Hermansson	(L-126535)243762	UPS
Micarea hedlundii		x	x	x			Sweden	Hermansson & Lundqvist	(L-134944)268147	UPS
Micarea hedlundii		x	x	x			Sweden	Hermansson	(L-102834)180173	UPS
Micarea hedlundii		x	x	x			Sweden	Hermansson	(L-88907)158452	UPS
Micarea hedlundii		x	x	x			Sweden	Hermansson	(L-93098)163181	UPS
Micarea hedlundii		x	x	x			Sweden	Hermansson	(L-167449)399397	UPS
Micarea hedlundii		x	x	x			Sweden	Hermansson	L-656364	UPS
Micarea hedlundii		x	x	x			Sweden	Hermansson	(L-173118)430000	UPS
Micarea hedlundii		x	x	x			Sweden	Hermansson	(L-173146)430028	UPS
Micarea hedlundii		x	x	x			Sweden	Hermansson	(L-107336)195363	UPS
Micarea hedlundii	x	x	x	x			Sweden	Hermansson	L-564753	UPS
Micarea hedlundii		x	x	x			Sweden	Hermansson	(L-173145)430027	UPS
Micarea hedlundii		x	x	x			Sweden	Hermansson	(L-093190)163318	UPS
Micarea hedlundii		x	x	x			Sweden	Forslund & Koffman	(L-158163) 348193	UPS
Micarea hedlundii		x	x	x			Sweden	Hermansson	L-564719	UPS
Micarea hedlundii		x	x	x			Sweden	Hermansson	(L-125376)241345	UPS
Micarea hedlundii		x	x	x			Sweden	Hermansson	(L-102727)180066	UPS
Micarea hedlundii		x	x	x			Sweden	Tibell	(L-005555)15842	UPS
Micarea hedlundii		x	x	x			Sweden	Hermansson	(L-102818)180157	UPS
Micarea hedlundii		x	x	x			Sweden	Nordin	(L-64018) 113114	UPS

Micarea hedlundii		x	x	x			Sweden	Muhr	L-602171	UPS
Micarea hedlundii		x	x	x			Sweden	Hermansson	(L-54952)91696	UPS
Micarea hedlundii		x	x	x			Finland	Pykälä	34470	H
Micarea hedlundii		x	x	x			Finland	Launis (Kantelinen)	67119	H
Micarea hedlundii		x	x	x			Finland	Launis (Kantelinen)	1510131	H
Micarea hedlundii		x	x	x			Finland	Pykälä	32808	H
Micarea hedlundii		x	x	x			Finland	Launis (Kantelinen)	109101	H
Micarea hedlundii		x	x	x			Finland	Launis (Kantelinen)	59132	H
Micarea hedlundii		x	x	x			Finland	Launis (Kantelinen)	35708	H
Micarea hedlundii		x	x	x			Norway	Haugan	11948	O
Micarea hedlundii		x	x	x			Norway	Haugan	11949	O
Micarea hedlundii		x	x	x			Norway	Haugan	11840	O
Micarea hedlundii		x	x	x			Finland	Pykälä	47685	H
Micarea hedlundii		x	x	x			Finland	Pykälä	47204	H
Micarea hedlundii		x	x	x			Finland	Pykälä	47183	H
Micarea hedlundii		x	x	x			Finland	Pykälä	47224	H
Micarea hedlundii		x	x	x			Finland	Pykälä	31826	H
Micarea hedlundii		x	x	x			Finland	Pykälä	47202	H
Micarea hedlundii		x	x	x			Russia	Himelbrant & Stepanchikova	Mets-01-10	H
Micarea hedlundii		x	x	x			Poland	Kukwa	14341	UGDA
Micarea hedlundii		x	x	x			Poland	Kukwa	14225	UGDA
Micarea hedlundii	x	x	x	x			Poland	Kukwa	15619	UGDA
Micarea hedlundii	x	x	x	x			Poland	Kukwa	15585	UGDA
Micarea hedlundii	x	x	x	x			Poland	Kukwa	15579	UGDA
Micarea hedlundii		x	x	x			Poland	Kukwa	15962	UGDA

Micarea isidioprasina	x		x		x		Poland	Kukwa	14030	UGDA
Micarea isidioprasina		x	x	x			Poland	Kukwa	13299	UGDA
Micarea isidioprasina		x	x	x			Poland	Kukwa	17493	UGDA
Micarea isidioprasina	x		x	x			Poland	Kukwa	14243	UGDA
Micarea isidioprasina	x		x	x			Poland	Kukwa	14358	UGDA
Micarea isidioprasina	x		x		x		Poland	Kukwa	14038	UGDA
Micarea isidioprasina		x	x			x	Poland	Kukwa	14112	UGDA
Micarea isidioprasina	x		x	x			Poland	Kukwa	14107	UGDA
Micarea isidioprasina	x		x			x	Poland	Kukwa	13418	UGDA
Micarea isidioprasina	x		x	x			Poland	Kukwa	17367a	UGDA
Micarea laeta	x		x		x		Germany	Printzen & students	TS2-99-1-6	FR
Micarea laeta	x		x	x			Germany	Printzen & students	TS2-88-19-16	FR
Micarea laeta	x		x		x		Sweden	Nordin, Sundin & Thor	(L-161769)365723	UPS
Micarea laeta	x		x		x		Sweden	Nordin	(L-179116)449462	UPS
Micarea laeta	x		x		x		Finland	Launis (Kantelinen)	59153a	H
Micarea laeta	x		x		x		Finland	Launis (Kantelinen)	1510131	H
Micarea laeta	x		x		x		Finland	Launis (Kantelinen)	59153	H
Micarea laeta	x		x		x		Finland	Launis (Kantelinen)	49151	H
Micarea laeta	x		x		x		Finland	Launis (Kantelinen)	59154	H
Micarea laeta	x		x		x		Finland	Launis (Kantelinen)	59155	H
Micarea laeta	x		x		x		Finland	Launis (Kantelinen)	49152	H
Micarea laeta	x		x		x		Finland	Launis (Kantelinen)	186152	H
Micarea laeta	x		x		x		Finland	Launis (Kantelinen)	269141	H

Micarea laeta	x		x		x		Finland	Launis (Kantelinen)	286151	H
Micarea laeta	x		x		x		Finland	Launis (Kantelinen)	1010133	H
Micarea laeta	x		x		x		Finland	Launis (Kantelinen)	1010134	H
Micarea laeta	x		x		x		Finland	Launis (Kantelinen)	1010135	H
Micarea laeta	x		x		x		Russia	Himelbrant & Stepanchikova	H9219948	H
Micarea laeta	x		x	x			Germany	Weber	FR-0267185	FR
Micarea melanobola	x		x	x			Germany	Printzen & students	TS3-13,-14	FR
Micarea melanobola	x		x	x			Germany	Printzen & students	TS3-12,-14	FR
Micarea melanobola	x		x	x			Germany	Printzen & students	TS3-13,-12	FR
Micarea melanobola	x		x	x			Germany	Printzen & students	TS3-5,-11	FR
Micarea melanobola	x		x	x			Germany	Printzen & students	TS3-3,-11	FR
Micarea melanobola	x		x	x			Germany	Printzen & students	TS3-1,-14	FR
Micarea melanobola	x		x	x			Germany	Printzen & students	TS2-88-18-9	FR
Micarea melanobola	x		x		x		Sweden	Westberg, Ekman, Hirschheydt	L-872026	UPS
Micarea melanobola	x		x		x		Sweden	Westberg, Ekman, Hirschheydt	L-872044	UPS
Micarea melanobola	x		x		x		Sweden	Westberg, Ekman, Hirschheydt	L-872146	UPS
Micarea melanobola	x		x		x		Sweden	Launis (Kantelinen)	L-949282	UPS
Micarea melanobola	x		x		x		Sweden	Hedlund	L-773781	UPS
Micarea melanobola	x		x		x		Finland	Pykälä	54151	H
Micarea melanobola	x		x	x			Finland	Launis (Kantelinen)	79133	H
Micarea melanobola	x		x		x		Finland	Launis (Kantelinen)	27123	H

Micarea melanobola	x		x		x		Finland	Launis (Kantelinen)	11014	H
Micarea melanobola	x		x		x		Finland	Launis (Kantelinen)	49141	H
Micarea melanobola	x		x		x		Finland	Launis (Kantelinen)	116152	H
Micarea melanobola	x		x		x		Finland	Launis (Kantelinen)	56151	H
Micarea melanobola	x		x		x		Finland	Launis (Kantelinen)	39151	H
Micarea melanobola	x		x		x		Finland	Launis (Kantelinen)	286152	H
Micarea melanobola	x		x		x		Finland	Launis (Kantelinen)	266151	H
Micarea melanobola	x		x		x		Finland	Launis (Kantelinen)	166151	H
Micarea melanobola	x		x	x			Poland	Kukwa	13740	UGDA
Micarea microareolata	x		x		x		Finland	Launis (Kantelinen)	59152	H
Micarea microareolata	x		x		x		Finland	Pykälä	47783	H
Micarea microareolata	x		x		x		Finland	Pykälä	47787	H
Micarea microareolata	x		x		x		Finland	Launis (Kantelinen)	59133	H
Micarea microareolata	x		x		x		Finland	Launis (Kantelinen)	89133	H
Micarea microareolata	x		x		x		Finland	Launis (Kantelinen)	186151	H
Micarea microareolata	x		x		x		Finland	Pykälä	47948	H
Micarea microareolata	x		x		x		Sweden	Launis (Kantelinen)	148131	H
Micarea microareolata	x		x		x		Sweden	Launis (Kantelinen)	148132	H
Micarea micrococca	x		x	x			Poland	Kukwa	17233	UGDA
Micarea micrococca	x	x	x	x			Germany	Schneider	FR-0263180	FR
Micarea micrococca	x		x	x			Germany	Printzen & students	TS2-88-4_3	FR

Micarea micrococca	x		x		x		Finland	Launis (Kantelinen)	299101	H
Micarea micrococca	x		x		x		Finland	Launis (Kantelinen)	238131	H
Micarea micrococca	x		x		x		Finland	Launis (Kantelinen)	1210131	H
Micarea micrococca	x		x	x			Finland	Launis (Kantelinen)	1210132	H
Micarea micrococca	x	x	x	x			Russia	Stepanchikova & Tagirdzhanova	KB-04-2013	H
Micarea micrococca	x		x		x		Sweden	Hermansson	10511	UPS
Micarea micrococca	x		x	x			Poland	Kukwa	15789	UGDA
Micarea microsorediata	(x)	x	x		x		Germany	Schön	FR-0221443	FR
Micarea microsorediata	x		x	x			Poland	Kukwa	17053	UGDA
Micarea microsorediata		x	x		x		Poland	Kukwa	7721	UGDA
Micarea microsorediata		x	x	x			Poland	Kukwa	13462	UGDA
Micarea microsorediata		x	x		x		Poland	Kukwa	17001a	UGDA
Micarea microsorediata	x		x	x			Poland	Kukwa	14350	UGDA
Micarea microsorediata		x	x		x		Poland	Kukwa	13420	UGDA
Micarea microsorediata	(x)		x		x		Poland	Kukwa	13321	UGDA
Micarea microsorediata		x	x		x		Poland	Kukwa	17592	UGDA
Micarea microsorediata	x		x	x			Poland	Kukwa	17641	UGDA
Micarea microsorediata		x	x	x			Poland	Kukwa	15789	UGDA
Micarea microsorediata		x	x		x		Poland	Kukwa	19991	UGDA
Micarea microsorediata		x	x		x		Poland	Kukwa	17379	UGDA

Micarea microsorediata		x	x		x		Poland	Kukwa	14002	UGDA
Micarea microsorediata		x	x		x		Poland	Kukwa	14000	UGDA
Micarea microsorediata	(x)		x		x		Poland	Kukwa	17546	UGDA
Micarea microsorediata		x	x		x		Poland	Kukwa	17040	UGDA
Micarea microsorediata		x	x	x			Poland	Kukwa	14012	UGDA
Micarea microsorediata		x	x		x		Poland	Kukwa	13778	UGDA
Micarea microsorediata		x	x		x		Poland	Kukwa	17032	UGDA
Micarea microsorediata		x	x		x		Poland	Kukwa	14045	UGDA
Micarea microsorediata		x	x	x			Poland	Kukwa	16994	UGDA
Micarea microsorediata		x	x		x		Poland	Kukwa	19839	UGDA
Micarea microsorediata			x		x		Poland	Kukwa	19212	UGDA
Micarea microsorediata		x	x		x		Poland	Kukwa	19849	UGDA
Micarea microsorediata	x		x		x		Poland	Kukwa	19850	UGDA
Micarea microsorediata	(x)		x		x		Poland	Kukwa	19219	UGDA
Micarea microsorediata		x	x		x		Poland	Kukwa	19801	UGDA
Micarea microsorediata		x	x		x		Poland	Kukwa	19800a	UGDA
Micarea nowakii	x	x	x	x			Germany	Schneider	FR-0263218	FR
Micarea nowakii	x		x	x			Germany	Printzen & students	TS3-17,-12	FR
Micarea nowakii	x		x	x			Germany	Printzen & students	TS3-25,-4	FR
Micarea nowakii	x		x	x			Germany	Printzen & students	TS4-56-11	FR
Micarea nowakii	x	x	x	x			Sweden	Blomberg	(L-195403)488087	UPS

Micarea nowakii	x	x	x	x			Sweden	Johansson	L-695951	UPS
Micarea nowakii	x	x	x	x			Sweden	Johansson	L-695952	UPS
Micarea nowakii	x	x	x	x			Sweden	Johansson	L-695948	UPS
Micarea nowakii	x	x	x	x			Sweden	Johansson	L-695950	UPS
Micarea nowakii	x	x	x	x			Sweden	Johansson	L-695953	UPS
Micarea nowakii	x	x	x	x			Sweden	Johansson	L-695954	UPS
Micarea nowakii	x	x	x	x			Sweden	Johansson	L-695947	UPS
Micarea nowakii	x	x	x	x			Sweden	Johansson	L-695956	UPS
Micarea nowakii	x	x	x	x			Sweden	Johansson	L-695949	UPS
Micarea nowakii	x	x	x	x			Sweden	Johansson	L-695955	UPS
Micarea nowakii	x	x	x	x			Sweden	Svensson	L-532777	UPS
Micarea nowakii	x	x	x	x			Finland	Launis (Kantelinen)	684	H
Micarea pauli	x		x		x		Poland	Kukwa	17240	UGDA
Micarea pauli	x		x	x			Poland	Kukwa	13308	UGDA
Micarea pauli		x	x		x		Poland	Kukwa	14101	UGDA
Micarea pauli	x		x		x	x	Poland	Kukwa	17621	UGDA
Micarea pauli	x		x		x		Poland	Kukwa	17227	UGDA
Micarea pauli	x		x		x		Poland	Kukwa	17544	UGDA
Micarea pauli		x	x		x		Poland	Kukwa	13345	UGDA
Micarea pauli	x		x	x			Poland	Kukwa	17619	UGDA
Micarea pauli		x	x		x	x	Poland	Kukwa	13194	UGDA
Micarea prasina	x		x	x			Germany	Schneider	FR-0263054	FR
Micarea prasina	x		x	x			Germany	Schneider	FR-0262859	FR
Micarea prasina	x		x	x			Germany	Schneider	FR-0262908	FR
Micarea prasina	x		x	x			Germany	Schneider	FR-0263057	FR
Micarea prasina	x		x	x			Germany	Schneider	FR-0263189	FR
Micarea prasina	x		x	x			Germany	Schneider	FR-0263200	FR
Micarea prasina	x		x	x			Germany	Schneider	FR-0263122	FR
Micarea prasina	x		x	x			Germany	Schneider	FR-0263077	FR

Micarea prasina	x		x	x			Germany	Printzen & students	TS2-88-10-6	FR
Micarea prasina	x		x	x			Sweden	Knutsson	L-720672	UPS
Micarea prasina	x		x	x			Sweden	Knutsson	L-720686	UPS
Micarea prasina	x		x	x			Sweden	Tibell	L-649274	UPS
Micarea prasina	x		x	x			Sweden	Tibell	L-774437	UPS
Micarea prasina	x		x	x			Sweden	Nordin	(L-58794)99147	UPS
Micarea prasina	x		x		x		Sweden	Svensson	(L-159411)351595	UPS
Micarea prasina	x		x		x		Sweden	Hermansson	(L-107292)195319	UPS
Micarea prasina	x		x		x		Sweden	Hermansson	L-663581	UPS
Micarea prasina	x		x		x		Sweden	Hermansson	(L-128035)248851	UPS
Micarea prasina	x		x	x			Sweden	Hermansson	(L-111495)205340	UPS
Micarea prasina	x		x		x		Sweden	Hermansson	(L-125626)241595	UPS
Micarea prasina	x		x	x			Sweden	Nordin	(L-131960)262417	UPS
Micarea prasina	x		x	x			Sweden	Nordin	(L-86954)155852	UPS
Micarea prasina	x		x		x		Sweden	Thor	(L-165410)388122	UPS
Micarea prasina	x		x	x			Finland	Pykälä	54351	H
Micarea prasina	x		x	x			Austria	Hafellner	43132	H
Micarea prasina	x		x	x			Finland	Launis (Kantelinen)	265101	H
Micarea prasina	x		x	x			Finland	Launis (Kantelinen)	229101	H
Micarea prasina	x		x	x			Finland	Launis (Kantelinen)	199105	H
Micarea prasina	x		x	x			Finland	Launis (Kantelinen)	59131	H
Micarea prasina	x		x	x			Finland	Launis (Kantelinen)	89131	H

Micarea prasina	x		x		x		Finland	Launis (Kantelinen)	89135	H
Micarea prasina	x		x	x			Russia	Kuznetsova & Stepanchikova	H9219946	H
Micarea prasina	x		x	x			Russia	Stepanchikova	Tuters-11-2015	H
Micarea prasina	x		x		x		Sweden	Hermansson	19157	UPS
Micarea prasina	x		x		x		Sweden	Hermansson	16222b	UPS
Micarea prasina	x		x		x		Sweden	Hermansson	6538	UPS
Micarea prasina	x		x		x		Sweden	Svensson	454	UPS
Micarea prasina	x		x	x			Poland	Wilk	UGDA-L-17377	UGDA
Micarea prasina	x		x	x			Poland	Kukwa	13733	UGDA
Micarea prasina	x		x	x			Poland	Kukwa	13720	UGDA
Micarea prasina	x		x	x			Poland	Kukwa	13373	UGDA
Micarea prasina	x		x	x			Poland	Kukwa	13387	UGDA
Micarea prasina	x		x	x			Poland	Kukwa	13408	UGDA
Micarea prasina	x		x		x		Poland	Kukwa	13393	UGDA
Micarea pseudomicrococca	x		x	x			Germany	Schneider	FR-0263076	FR
Micarea pseudomicrococca	x		x	x			Sweden	Forslund & Koffman	(L-158384)348414	UPS
Micarea pseudomicrococca	x		x	x			Germany	Printzen & students	TS3-15,-7	FR
Micarea pseudomicrococca	x		x	x			Germany	Printzen & students	TS3-15,-8	FR
Micarea pseudomicrococca	x		x	x			Germany	Printzen & students	TS3-42,-16	FR
Micarea pseudomicrococca	x		x	x			Germany	Printzen & students	TS3-44,-13	FR
Micarea pseudomicrococca	x		x	x			Germany	Printzen & students	TS3-46,-15	FR
Micarea pseudomicrococca	x		x	x			Germany	Printzen & students	TS4-20,-12	FR
Micarea pseudomicrococca	x		x	x			Germany	Printzen & students	TS4-20,-4	FR

Micarea pseudomicrococca	x		x	x			Germany	Printzen & students	TS2-88-6	FR
Micarea pseudomicrococca	x		x	x			Germany	Printzen & students	TS2-88-14-4	FR
Micarea pseudomicrococca	x		x	x			Germany	Printzen & students	TS2-88-18-7	FR
Micarea pseudomicrococca	x		x	x			Germany	Printzen & students	TS2-88-17	FR
Micarea pseudomicrococca	x		x		x		Finland	Pykälä	53268	H
Micarea pseudomicrococca	x		x		x		Finland	Pykälä	53258	H
Micarea pseudomicrococca	x		x		x		Finland	Pykälä	53278	H
Micarea pseudomicrococca	x		x		x		Finland	Launis (Kantelinen)	59151	H
Micarea pseudomicrococca	x		x	x			Finland	Launis (Kantelinen)	89132	H
Micarea pseudomicrococca	x		x		x		Finland	Launis (Kantelinen)	258131	H
Micarea pseudomicrococca	x		x		x		Finland	Pykälä	47579	H
Micarea pseudomicrococca	x		x		x		Finland	Pykälä	47574	H
Micarea pseudomicrococca	x	x	x		x		Russia	Stepanchikova & Tagirdzhanova	KB-06-2013	H
Micarea pseudomicrococca	x		x	x			Sweden	Forslund & Koffman	790	UPS
Micarea pseudotsugae	x		x		x		Netherlands	van den Boom	holotyyppi, no number, collect date 5/5/2019	UGDA
Micarea pusilla	x		x	x			Germany	Printzen & students	TS3-34,-17	FR
Micarea pusilla	x		x	x			Germany	Printzen & students	TS3-34,-13	FR
Micarea pusilla	x		x	x			Germany	Printzen & students	TS4-20,-10	FR
Micarea pusilla	x		x	x			Finland	Launis (Kantelinen)	101035	H

Micarea pusilla	x		x	x			Czech	Vondrák	14632	PRA
Micarea pusilla	x		x	x			Czech	Malíček	9590	Hb Malíček
Micarea pusilla	x		x	x			Czech	Vondrák	14634	PRA
Micarea pusilla	x		x	x			Czech	Vondrák	14633	PRA
Micarea pusilla	x		x	x			Czech	Malíček	9903	Hb Malíček
Micarea pusilla	x		x	x			Czech	Malíček	9636	Hb Malíček
Micarea pusilla	x		x	x			Czech	Vondrák	16643	PRA
Micarea pusilla	x		x	x			Czech	Malíček	12017	Hb Malíček
Micarea pusilla	x		x		x		Finland	Launis (Kanteline n)	1010136	H
Micarea pusilla	x		x		x		Finland	Launis (Kanteline n)	1010137	H
Micarea pusilla	x		x	x			Russia	Vondrák	14668	PRA
Micarea pusilla	x		x	x			Russia	Malíček	10449	Hb Malíček
Micarea pusilla	x		x		x		Russia	Himelbran t & Stepanchi kova	H9220216	H
Micarea soralifera		x	x	x			German y	Schneider	FR-0262786	FR
Micarea soralifera	x		x	x			Poland	Kukwa	13001	UGDA
Micarea soralifera		x	x		x		Poland	Kukwa	19210b	UGDA
Micarea soralifera		x	x		x		Poland	Kukwa	15850	UGDA
Micarea soralifera	x		x	x			Poland	Kukwa	15903	UGDA
Micarea soralifera		x	x		x		Poland	Kukwa	17615	UGDA
Micarea soralifera	x	x	x	x			Poland	Kukwa	13000	UGDA
Micarea soralifera		x	x		x		Poland	Kukwa	15626	UGDA
Micarea soralifera	(x)	x	x		x		Poland	Kukwa	17674	UGDA
Micarea soralifera	x		x	x			Poland	Kukwa	17650	UGDA
Micarea soralifera	x	x	x	x			Poland	Kukwa	13469	UGDA
Micarea soralifera	x	x	x	x			Poland	Kukwa	15572	UGDA
Micarea soralifera	(x)	x	x	x			Poland	Kukwa	17202	UGDA
Micarea soralifera	x	x	x	x			Poland	Kukwa	17211	UGDA

Micarea soralifera	x	x	x	x			Poland	Kukwa	17258	UGDA
Micarea soralifera	x	x	x	x			Poland	Kukwa	17261	UGDA
Micarea soralifera	x	x	x	x			Poland	Kukwa	12939	UGDA
Micarea soralifera	x	x	x	x			Poland	Kukwa	12663	UGDA
Micarea soralifera	x	x	x	x			Poland	Kukwa	12797	UGDA
Micarea soralifera	x	x	x	x			Poland	Kukwa	12949	UGDA
Micarea soralifera	x	x	x	x			Poland	Kukwa	12969	UGDA
Micarea soralifera	x	x	x	x			Poland	Kukwa	12999	UGDA
Micarea soralifera	x	x	x	x			Poland	Kukwa	5257	UGDA
Micarea soralifera		x	x	x			Poland	Kukwa	1504	UGDA
Micarea soralifera	x	x	x	x			Poland	Kukwa	15624	UGDA
Micarea soralifera		x	x	x			Poland	Kukwa	12473	UGDA
Micarea soralifera	x	x	x	x			Poland	Kukwa	12722	UGDA
Micarea soralifera	x	x	x	x			Poland	Kukwa	12863	UGDA
Micarea soralifera	x	x	x	x			Poland	Kukwa	14176	UGDA
Micarea soralifera	x	x	x	x			Poland	Kukwa	14154	UGDA
Micarea soralifera	x	x	x		x		Poland	Kukwa	15791	UGDA
Micarea soralifera	(x)	x	x	x			Poland	Kukwa	15938	UGDA
Micarea soralifera	x	x	x	x			Poland	Kukwa	15924	UGDA
Micarea soralifera	x	x	x	x			Poland	Kukwa	15900	UGDA
Micarea soralifera	x	x	x	x			Poland	Kukwa	15906	UGDA
Micarea soralifera	x	x	x	x			Poland	Kukwa	17490	UGDA
Micarea soralifera	x	x	x	x			Poland	Kukwa	17464	UGDA
Micarea soralifera		x	x	x			Poland	Kukwa	17503	UGDA
Micarea soralifera	x	x	x	x			Poland	Kukwa	17518	UGDA
Micarea soralifera		x	x	x			Poland	Kukwa	17491	UGDA
Micarea soralifera		x	x		x		Poland	Kukwa	17492	UGDA
Micarea soralifera		x	x	x			Poland	Kukwa	17488	UGDA
Micarea soralifera	x	x	x	x			Poland	Kukwa	13753	UGDA

Micarea soralifera	(x)	x	x	x			Poland	Kukwa	13480	UGDA
Micarea soralifera		x	x	x			Poland	Kukwa	13493	UGDA
Micarea soralifera	x	x	x	x			Poland	Kukwa	14037	UGDA
Micarea soralifera	x	x	x	x			Poland	Kukwa	13732	UGDA
Micarea soralifera	x	x	x	x			Poland	Kukwa	13774	UGDA
Micarea soralifera	x	x	x	x			Poland	Kukwa	13571	UGDA
Micarea soralifera	x	x	x	x			Poland	Kukwa	13270	UGDA
Micarea soralifera		x	x	x			Poland	Kukwa	13221	UGDA
Micarea soralifera	x	x	x	x			Poland	Kukwa	14020	UGDA
Micarea soralifera		x	x	x			Poland	Kukwa	13398	UGDA
Micarea soralifera	x	x	x	x			Poland	Kukwa	13959	UGDA
Micarea soralifera		x	x	x			Poland	Kukwa	13764	UGDA
Micarea soralifera	(x)	x	x		x		Poland	Kukwa	17350	UGDA
Micarea soralifera		x	x	x			Sweden	Nordin	L-909820	UPS
Micarea soralifera		x	x		x		Sweden	Westberg, Ekman, Hirschheydt	L-872084	UPS
Micarea soralifera		x	x	x			Sweden	Westberg, Ekman, Hirschheydt	L-790652	UPS
Micarea soralifera	x	x	x	x			Sweden	Nordin	L-797384	UPS
Micarea soralifera	x	x	x	x			Sweden	Westberg, Ekman, Hirschheydt	L-790650	UPS
Micarea soralifera	x	x	x	x			Sweden	Westberg & Johannesson	L-942546	UPS
Micarea soralifera		x	x	x			Finland	Launis (Kantelinen)	3075	H
Micarea tomentosa	x	x	x	x			Poland	Kukwa	15963	UGDA
Micarea tomentosa		x	x	x			Germany	Schneider	FR-0263200	FR
Micarea tomentosa		x	x		x		Poland	Kukwa	17555	UGDA
Micarea tomentosa		x	x	x			Poland	Kukwa	5096	UGDA
Micarea tomentosa		x	x	x			Poland	Kukwa	5122	UGDA

Micarea tomentosa	x	x	x	x			Poland	Kukwa	13352	UGDA
Micarea tomentosa	x	x	x	x			Poland	Kukwa	14188	UGDA
Micarea tomentosa	x	x	x	x			Sweden	Nordin	(L-113300)210588	UPS
Micarea tomentosa		x	x	x			Sweden	Svensson	(L-171669)411893	UPS
Micarea tomentosa	x	x	x	x			Sweden	Hermansson	(L-88916)158461	UPS
Micarea tomentosa		x	x	x			Finland	Launis (Kantelinen)	2431	H
Micarea tomentosa		x	x	x			Finland	Launis (Kantelinen)	2435	H
Micarea tomentosa		x	x	x			Finland	Launis (Kantelinen)	2437	H
Micarea tomentosa		x	x	x			Finland	Pykälä	49725	H
Micarea tomentosa		x	x	x			Finland	Launis (Kantelinen)	29151	H
Micarea tomentosa		x	x	x			Finland	Pykälä	55572	H
Micarea tomentosa		x	x	x			Sweden	Delin	(L-101264)177765	UPS
Micarea tomentosa		x	x	x			Russia	Muchnik	L-14975	LE
Micrea viridileprosa		x	x	x			Sweden	Thor	(L-200234)497493	UPS
